# Persistent CMV pneumonitis in HIV infection: a case report

**DOI:** 10.1186/s12879-023-08848-y

**Published:** 2023-11-29

**Authors:** Abbye W. Frederick, Ellen Kitchell, Clare McCormick-Baw, Vishal Kukkar, Mamta K. Jain

**Affiliations:** Division of Infectious Diseases & Geographic Medicine, University of Texas Southwestern, Dallas, TX USA

**Keywords:** CMV pneumonitis, HIV, Case report

## Abstract

We present a rare case of pathology-proven CMV pneumonitis in a patient with HIV infection after presenting with cough and fever. This presentation was complicated by recurrence of symptoms after treatment in the setting of continued uncontrolled HIV infection. This case raised the importance of further discussion regarding best treatment guidelines for CMV pneumonitis for patients with HIV.

## Introduction

DNAemia due to cytomegalovirus (CMV) infection is common in people living with human immunodeficiency virus (HIV) and is frequently detected in the blood of those with low CD4 counts [1]. Treatment for CMV DNAemia in HIV without end-organ disease is not recommended per the Department of Health and Human Services guidelines due to limited benefits and risk of harm [2]. People with HIV can develop symptomatic CMV retinitis and colitis which have their respective established treatment recommendations [3]. While CMV pneumonitis is a common complication in the transplant recipient population, it is an atypical entity in those with HIV. Cases and small cohorts of patients described previously in the literature were limited by varying approaches to the best diagnostic tests to use to establish CMV lung infection as well as variable lengths of treatment [4–8]. No established guidelines for treatment duration for CMV pneumonitis in HIV infection currently exist due to its rarity.

## Case presentation

A 39-year-old male was hospitalized for one month at an outside hospital with acute respiratory failure which required high flow oxygen supplementation. He was diagnosed with HIV with a CD4 cell count of 36 cells/µL. His risk factors included sex with men and women. Patient was not vaccinated for severe acute respiratory syndrome coronavirus 2 (SARS-CoV-2). He was also diagnosed with CMV DNAemia of 200,000 copies/mL in plasma and presumed *Pneumocystis jirovecii* (PJP) pneumonia given consistent imaging findings and a positive beta-D-glucan result. Sputum *Pneumocystis* Calcofluor white smear was negative, and the patient deferred bronchoscopy. He was treated for CMV DNAemia with intravenous ganciclovir for 3–4 weeks before transitioning to oral valganciclovir. He was also treated for PJP pneumonia with steroids and trimethoprim-sulfamethoxazole until developing hyperkalemia and transitioned to pentamidine to complete treatment. He was started on antiretroviral therapy (ART) with bictegravir/emtricitabine/tenofovir alafenamide. He was discharged on ART, valganciclovir, and prophylactic dapsone for PJP pneumonia prevention.

**Fig. 1:**
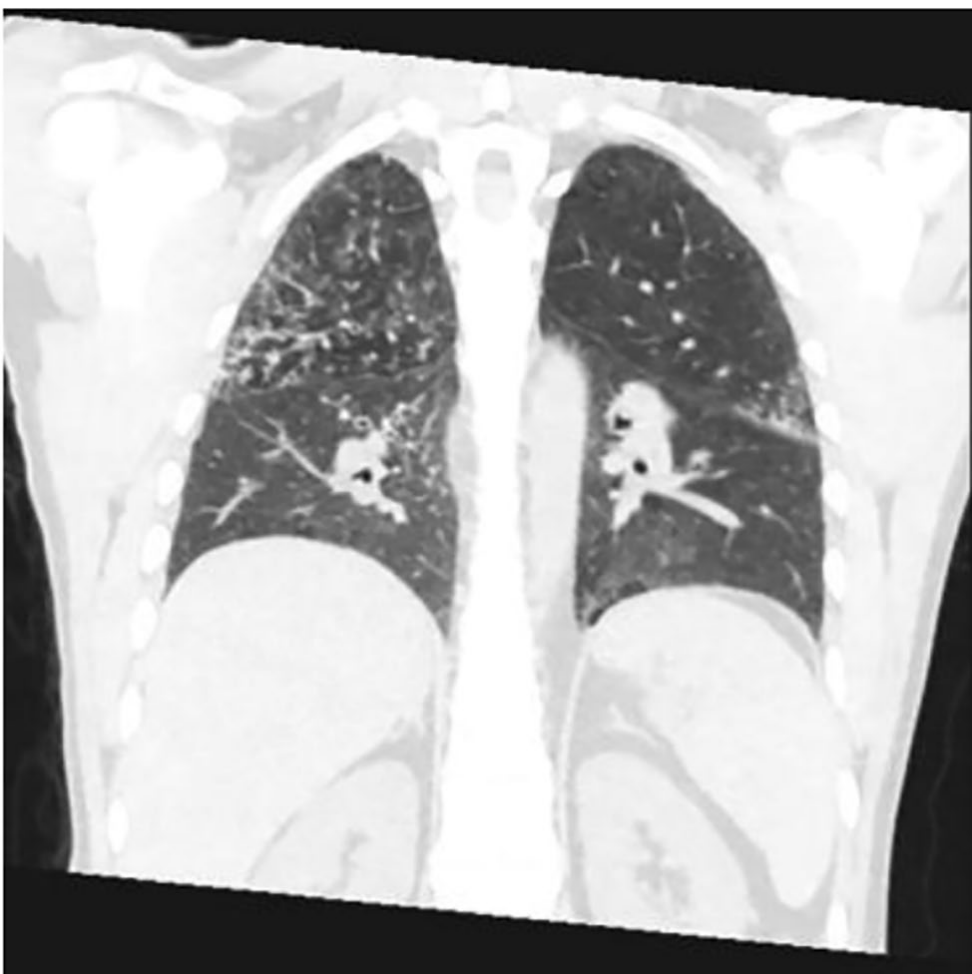
Coronal CT image shows bilateral peri-broncho-vascular nodules and ground glass opacities

**Fig. 2:**
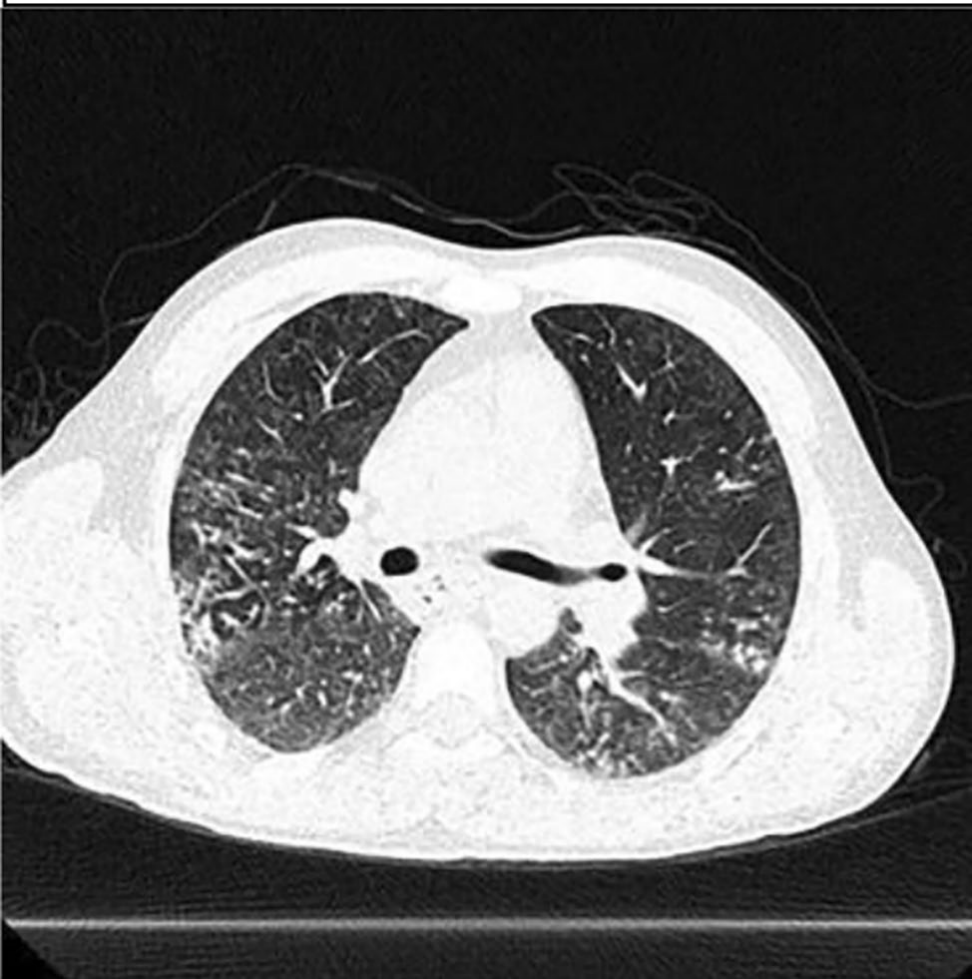
Axial CT image demonstrates nodules in peripheral aspects of right upper and left upper lobes

**Fig. 3:**
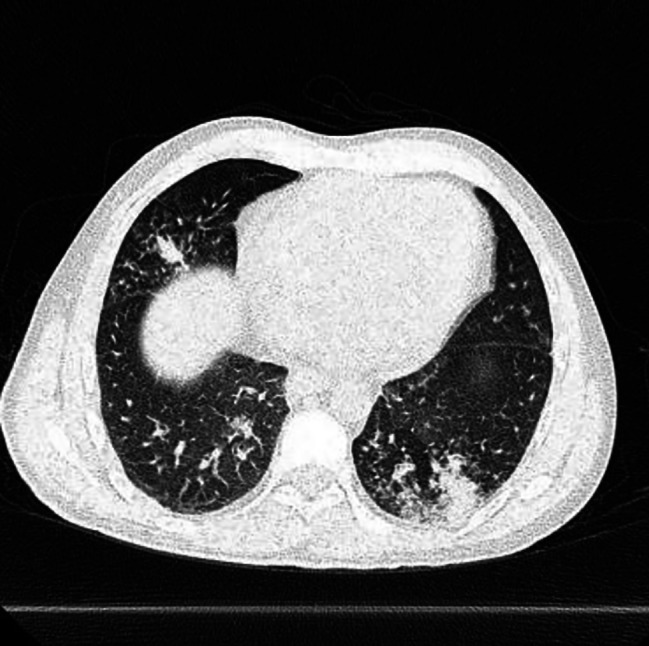
Axial CT image demonstrates nodules and consolidative opacities in right middle lobe and posterior left lung base

Four months after discharge from the outside hospital, the patient presented to the emergency department in the summer of 2022 with a dry cough for 3 weeks accompanied by intermittent fever, chills, night sweats, dysphagia, and diarrhea. He also had unintentional weight loss of 15 pounds over the last month. He had not been taking his medications for the last month and had been lost to follow up. Patient was raised in Mexico, but had no other recent travel, animal exposures, or current partners. On physical examination, his temperature was 37.8 C°, heart rate of 128 beats per minute, and a respiration rate of 26 breaths per minute with pulse oxygenation of 94%. The patient was diaphoretic but in no acute distress. Other findings were notable for oropharyngeal white patches, bilateral coarse breath sounds, and multiple flesh colored umbilicated papules on the left thorax. The exam was negative for hepatosplenomegaly or lymphadenopathy. On admission, chest x-ray revealed an ill-defined focal hazy opacity of the right lateral middle lung with a computerized tomography (CT) scan subsequently showing extensive areas of bilateral tree-in-bud infiltrate with scattered ground glass and consolidative opacities (Figs. 1, 2 and 3). Labs confirmed advanced HIV with CD4 of 6 cells/µL and a HIV viral load of > 800,000 copies/mL. He was found to be positive for SARS-CoV-2 by polymerase chain reaction (PCR). Plasma CMV PCR was elevated at 3.9 million copies/mL.

**Fig. 4:**
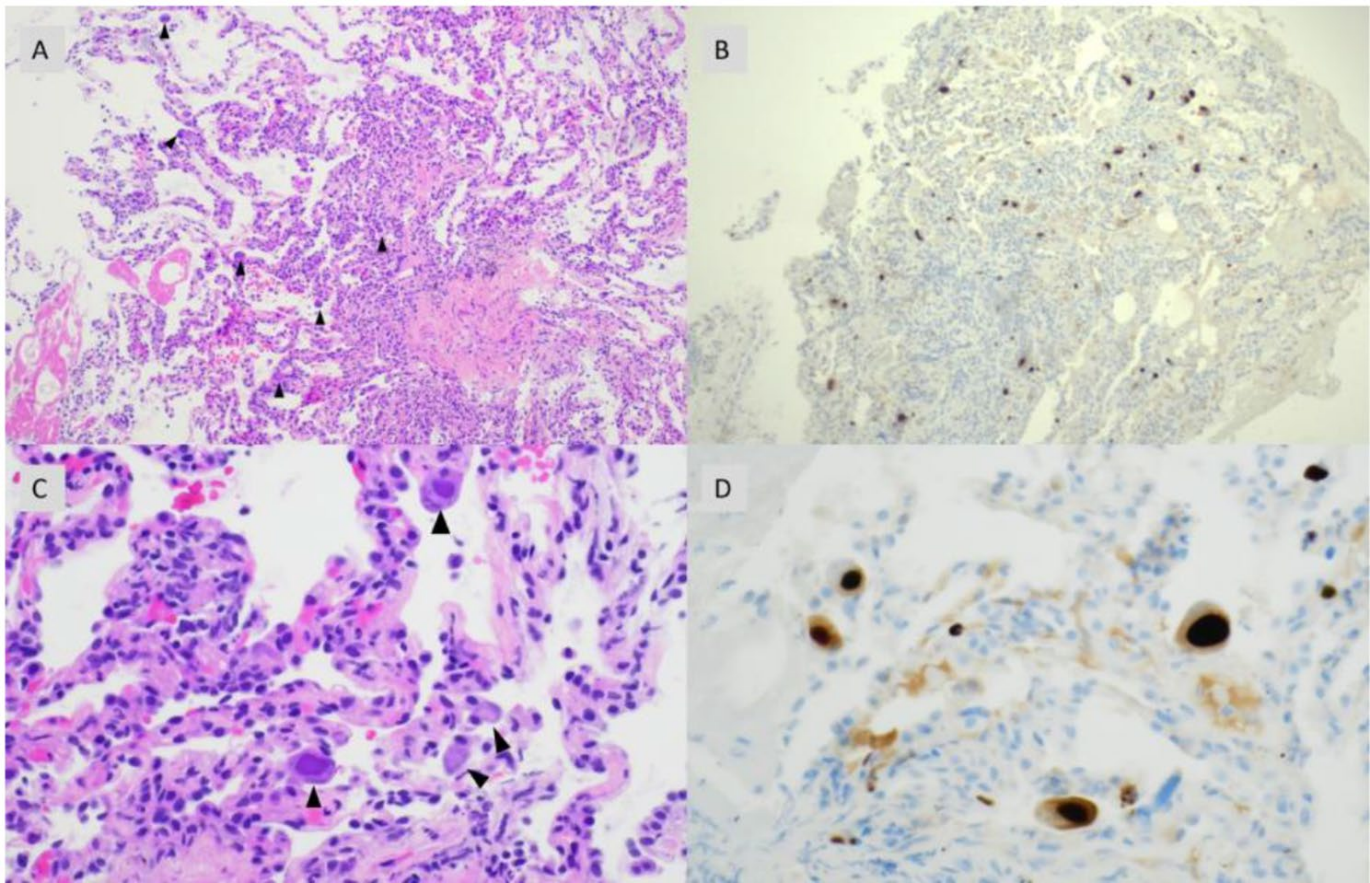
Histopathologic and immunohistochemical evaluation of transbronchial biopsy. **A**) Fragment of lung tissue with scattered enlarged cells (small arrowheads) with cytologic features diagnostic for CMV infection in a background of pneumonitis (100x magnification, H&E). **B**) Immunohistochemical confirmation using antibodies to CMV that are binding to enlarged cells (strong nuclear staining) scattered throughout the biopsy (100x magnification, CMV IHC). **C**) Multiple enlarged cells (arrowheads), some with characteristic nuclear inclusions in a background of reactive and inflamed lung parenchyma (400x magnification, H&E). **D**) CMV immunohistochemical staining with strong positivity in the nuclei and scattered positivity in the cytoplasm of the same cells (400x magnification, CMV IHC)

Coronavirus disease 2019 (COVID-19) treatment was deferred as the patient presented with subacute symptoms for several weeks prior to arrival without initial hypoxemia. The patient was started on empiric typical and atypical bacterial pneumonia treatment with vancomycin, cefepime, and doxycycline. He also started PJP treatment with trimethoprim-sulfamethoxazole. Additionally, the patient was started on fluconazole for presumed *Candida* esophagitis. However, during his admission, the patient developed worsening oxygen requirements and persistent fevers. CT angiogram did not show evidence of pulmonary embolism. C-reactive protein was elevated at 1.7 mg/dL and ferritin was 4,003 ng/mL. Bronchoalveolar lavage (BAL) was performed including viral culture which was positive for CMV. SARS-CoV-2 specific testing was not performed on the BAL fluid, and it is not routinely recovered from the cell lines used for viral culture at the reference laboratory. AFB culture was also performed on the BAL which later grew *Mycobacterium avium* complex (MAC). Transbronchial biopsies taken demonstrated pneumonitis with numerous enlarged, virally infected cells with both cytoplasmic and large nuclear inclusions. These findings were diagnostic of CMV pneumonitis (Fig. 4). Immunostaining confirmed numerous, scattered positive cells for CMV (Fig. 4B and D) whereas stains for acid fast bacilli and fungi were negative. The patient was initiated on intravenous ganciclovir for CMV pneumonitis. Dilated ophthalmologic exam did not reveal retinitis. The patient’s fevers, dyspnea, and cough resolved over the next seven days, and he was transitioned to oral valganciclovir. Of note, the MAC isolated on BAL was not treated due to patient’s improvement on other therapy.

**Fig. 5:**
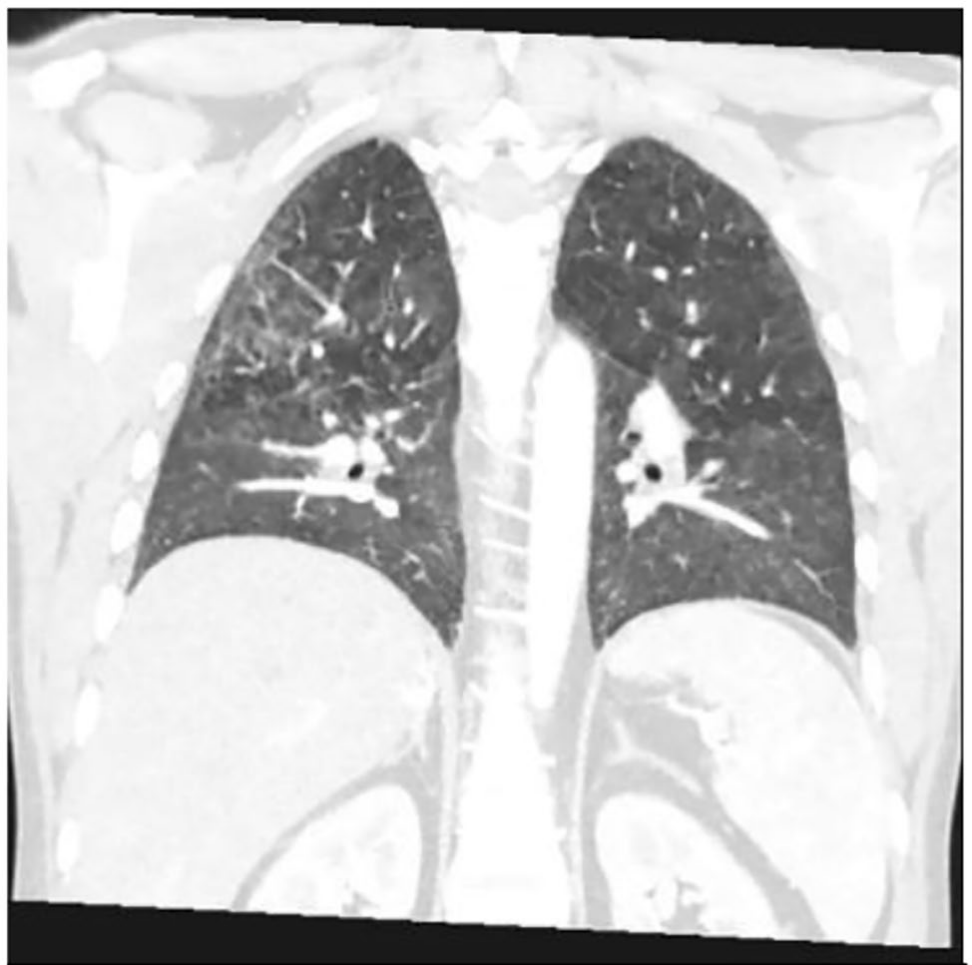
Coronal CT image obtained 3 months later with resolution of majority of the nodular opacities with residual ground glass opacities in right upper lobe

The patient was prescribed a 21-day course of valganciclovir. At outpatient follow up, he reported doing well and was finishing his CMV therapy. On that visit, he was reinitiated on ART with abacavir/dolutegravir/lamivudine. He presented again 2 months after initial presentation with dyspnea and fevers, requiring readmission to the hospital. A repeat CT revealed resolution of the majority of nodular opacities and residual ground glass opacities (Fig. 5), but his CMV PCR showed a plasma level of 5 million copies/mL. Patient’s SARS-CoV-2 by PCR testing was persistently positive, which seemed to indicate inability to clear the virus versus a continued active infection. He was briefly treated with intravenous foscarnet given initial concern for ganciclovir resistance; however, the mutational analysis for ganciclovir, cidofovir, and foscarnet resistance was negative with codons 457–630 of UL97 gene and codons 393–1000 of UL54 gene sequencing. Repeat CD4 was < 10 cells/µL and HIV viral load was 6 million copies/mL concerning for non-adherence to ART. The patient was switched back to intravenous ganciclovir and then to oral valganciclovir with symptomatic improvement.

## Discussion and conclusions

Patients with CMV pneumonitis have a clinical presentation of cough, fever, and hypoxia along with diffuse pulmonary infiltrates on imaging, nonspecific findings which can be seen with other infections [9]. A transbronchial biopsy is critical to identifying CMV inclusions on lung tissue or cytology evaluation of BAL material with evidence of viral cytologic effect. CMV PCR or culture on plasma or BAL alone is not adequate for diagnosis [9]. Cytology from BAL fluid for phenotypic findings is especially helpful when biopsy deemed too high risk [10]. Given the significant risks of end-organ disease in patients with HIV, the risk predictor, CMV elispot, could conceivably be considered for this population as it has been for the transplant population [11]. The treatment for CMV infections is ganciclovir followed by valganciclovir of variable durations. Treatment with ganciclovir can have significant toxicities, most commonly pancytopenia. Although CMV pneumonitis in HIV is rare, the patient’s symptoms, imaging, and pathology were consistent with CMV pneumonitis.

Our case differs from other reports, as we witnessed what appeared to be multiple recurrences. Our patient did receive CMV treatment before arrival at our facility for DNAemia; however, his original respiratory symptoms may have been due to PJP pneumonia. A singular etiology cannot be identified because he improved with treatment for both CMV and PJP. Theoretically his presentation at our institution may have been his first relapse of CMV. While he additionally had the confounder of a positive SARS-CoV-2 test, it did not appear to explain the timeline of the patient’s reported symptoms. Though improved initially on CMV treatment, our patient relapsed again soon after treatment ended at our facility. One possibility considered was resistance to ganciclovir; however, testing for CMV resistance was negative. An alternative hypothesis was immune reconstitution inflammatory syndrome (IRIS) given the time course of symptoms was 2 months after the initiation of ART. However, his viral load had increased since his last admission, consistent with non-adherence to ART.

Regardless of whether the patient was on ART or non-adherent, his CD4 cell count was low, raising the question of the need for prolonged treatment or maintenance therapy after induction. Providing CMV suppression until some level of immune reconstitution has occurred prior to stopping treatment may be needed in patients with AIDS with severely low CD4 cell count. It is clear our patient did not respond to a short course of ganciclovir and a longer treatment may have prevented re-admission. However, this should be individualized to the patient. In this case ART non-adherence was likely the driver of relapse and not the duration of CMV treatment.

## Data Availability

All data generated or analyzed during this study are included in this published article.

## Abbreviations

CMV: Cytomegalovirus
HIV: Human immunodeficiency virus
PJP: *Pneumocystis jirovecii*
ART: Antiretroviral therapy
CT: Computerized tomography
SARS CoV-2: Severe acute respiratory syndrome coronavirus 2
PCR: Polymerase chain reaction
BAL: Bronchoalveolar lavage
IRIS: Immune reconstitution inflammatory syndrome
MAC: *Mycobacterium avium* complex
